# Mental health and life satisfaction among nurses: a cross-sectional study

**DOI:** 10.1192/j.eurpsy.2022.1466

**Published:** 2022-09-01

**Authors:** A. Kchaou, A. Abbes, A. Hrairi, R. Masmoudi, F. Khrifech, K. Hammami, M.L. Masmoudi, J. Masmoudi, M. Hajjaji

**Affiliations:** 1 Hedi Chacker Hospital, Occupational Medicine, Sfax, Tunisia; 2 Hospital Hédi Chaker, Sfax, Sfax, Tunisia; 3 Hospital university of HEDI CHAKER, Psychiatry A Department, Sfax, Tunisia

**Keywords:** Nurses, general health, life satisfaction, mental health

## Abstract

**Introduction:**

Nursing is highly demanding and stressful profession. Therefore, maintaining and improving psychological wellbeing among nurses seems essential to have a better life satisfaction and a better productivity.

**Objectives:**

The aim of this study was to assess the relationship between mental health and life satisfaction among nurses.

**Methods:**

The study was conducted in a group of active nurses from Hedi Chaker hospital in Sfax- Tunisia. Standardized questionnaires were used, including the general health questionnaire (GHQ-28) and the satisfaction with life scale (SWLS).

**Results:**

A total of 100 (males = 40; females = 60) nurses participated in this study. The mean age was 50.20 ±7.20 years. The average of job tenure was 25.25 ± 9.70 years. The majority of participants (66.7%) reported having chronic diseases. Rotating shifts work was noted in 72.50% of cases. Average scores for the GHQ-28 and the SWLS were respectively 30.66 ± 11.07 and 21.61± 6.23. The presence of chronic conditions was associated with psychological distress (higher GHQ-28 scores) (p = 0.01). Life satisfaction score was positively correlated with age (r= 0.29, p= 0.023), whereas it was negatively correlated with GHQ-28 scores (r= -0.36, p= 0.01). Low life satisfaction (SWLS scores between 5 and 14) was significantly associated with three domains of the GHQ-28: somatic symptoms (p = 0.008), anxiety (p = 0.001) and social dysfunction (p = 0.01).

**Conclusions:**

According to our study, low life satisfaction was associated with psychological disorder. Hence, nurses need support and subsequent interventions in order to improve psychological wellbeing and life-satisfaction.

**Disclosure:**

No significant relationships.

